# Reliability and validity of the short questionnaire to assess health-enhancing physical activity (SQUASH) in patients after total hip arthroplasty

**DOI:** 10.1186/1471-2474-9-141

**Published:** 2008-10-17

**Authors:** Robert Wagenmakers, Inge van den Akker-Scheek, Johan W Groothoff, Wiebren Zijlstra, Sjoerd K Bulstra, Johan WJ Kootstra, GC Wanda Wendel-Vos, Jos JAM van Raaij, Martin Stevens

**Affiliations:** 1 Department of orthopedics, university medical center Groningen, university of Groningen, the Netherlands; 2 Department of health sciences, university medical center Groningen, university of Groningen, the Netherlands; 3 Center for human movement sciences, university medical center Groningen, university of Groningen, the Netherlands; 4 Center for prevention and health services research, national institute for public health and the environment, Bilthoven, the Netherlands; 5 Department of orthopedics, martini hospital, Groningen, the Netherlands

## Abstract

**Background:**

Despite recognized benefits of regular physical activity on musculoskeletal fitness as well as general health, little is known about the physical activity behavior of patients after Total Hip Arthroplasty (THA). So far, no physical activity questionnaire has been validated in this category of patients. As the Short Questionnaire to Assess Health-enhancing physical activity (SQUASH) has been shown to be a fairly reliable and valid tool to gauge the physical activity behavior of the general Dutch adult population, we measured the reliability and relative validity of this tool in patients after THA.

**Methods:**

44 patients (17 men and 27 women, mean age 71 ± 8 years) completed the SQUASH twice with an in-between period of 2 to 6 weeks (mean 3.7). Reliability was determined by calculating the Spearman correlation coefficient between the activity scores of the separate questions as well as the total activity scores from both administrations. Additionally, a Bland & Altman analysis was performed for the total activity scores. Relative validity was determined using the Actigraph™ accelerometer, worn by 39 patients (15 men and 24 women, mean age 70 ± 8 years) for a 2-week period following the second questionnaire, as a criterion measure.

**Results:**

Spearman's correlation coefficient for overall reliability was 0.57. It varied between 0.45 and 0.90 for the separate questions. No systematic biases between readings were found. The Spearman correlation between Actigraph™ readings and total activity score was 0.67. It was 0.56 for total minutes of activity, 0.20 for time spent in light intensity activity, 0.40 for moderate activity and 0.35 for vigorous activity. Systematic bias was found between the SQUASH and the Actigraph™.

**Conclusion:**

The SQUASH can be considered to be a fairly reliable tool to assess the physical activity behavior of patients after THA. Validity was found to be comparable with those of other questionnaires, and as it is short and easy to fill in, it may prove to be a useful tool to assess physical activity in this particular subset of the population. However, the considerable systematic bias found in this study illustrates the need for further analysis of the validity of the SQUASH.

## Background

There is a growing awareness in Western society of the importance of physical activity for general health. Regular physical activity has proven to be effective in the prevention of several chronic conditions as well as the enhancement of musculoskeletal fitness, and is linked to a reduction in all-cause mortality [1-4]. A lack of physical activity is also considered to be an important burden on public health [5]. For these reasons, national and international guidelines have been developed recommending 30 minutes or more of moderate-intensity physical activity at least five days per week, or vigorous-intensity physical activity for a minimum of 20 minutes at least three days per week [6-8]. Furthermore, every adult is advised to perform activities that maintain or increase muscular strength and endurance at least twice each week [7]. Additionally, older adults are also advised to engage in activities that maintain or increase flexibility and for those at risk for falls in exercises that maintain and improve balance [8].

In order to assess physical activity at a population level, self-reported questionnaires are the most commonly practical tools employed [9]. The Short Questionnaire to Assess physical activity (SQUASH) [10] is an example of such a questionnaire [see additional file 1]. It was developed in the Netherlands and has been validated using an accelerometer. The scores on the SQUASH are considered to be sufficiently reliable and valid to measure the level of physical activity of a healthy adult population [10]. Nowadays it is used by government agencies to monitor physical activity of the Dutch population as well as compliance with guidelines for health-enhancing physical activity. As such, the SQUASH has provided insight into the physical activity behavior of the general Dutch adult population.

However, so far little is known about the physical activity behavior of an important and growing subset of the population: patients after total hip arthroplasty (THA). Total hip arthroplasty has become the preferred treatment for advanced osteoarthrosis of the hip and is one of the most frequently performed procedures in orthopedic surgery, with 22,500 THAs performed in the Netherlands in 2005 [11] and 202,500 in the United States in 2003 [12]. In the coming decades these numbers are expected to increase dramatically due to the projected growth of the older population and expanding indications [12-15].

In light of the beneficial effects of physical activity on health and musculoskeletal fitness, more insight into the physical activity behavior of patients after THA is needed. The SQUASH might be a useful tool towards providing this information. However, because it has been shown that self-reports can be inherently biased [16], it is important to assess a questionnaire's reliability and validity for every population in which it will be used [17]. As this has not been determined in the population of patients after THA, we assessed the reliability and validity of the scores on the SQUASH as a measure of the physical activity behavior in this specific subset of the general population.

## Methods

### Study population

The study population was randomly selected from a larger cohort of patients which was prospectively formed to study the physical activity behavior of patients one year after THA. This cohort consisted of patients who had undergone primary THA at University Medical Center Groningen or Martini Hospital Groningen. Selected patients were contacted by mail or phone and asked to participate in this study. From the 86 contacted patients, 44 were enrolled in the reliability study and 39 patients also in the validation study. The remaining patients were not willing to participate for various reasons. These patients did not show any differences in main characteristics (age, gender) compared to the patients in the study population. The study took place from March 2007 to September 2007. In this period we did not observe large differences in weather conditions between measurements, which could have influenced physical activity behavior.

The study was executed in accordance with the regulations of the Medical Ethical Board of University Medical Center Groningen. Written informed consent was obtained from all patients.

### Study design

As part of the prospective study, all patients were sent a questionnaire with an explanatory letter one year after THA. This self-administered questionnaire contained the SQUASH as well as some demographic questions. After completion and return of the questionnaire, those patients who were enrolled in the reliability study (reliability group) completed the SQUASH for a second time 2 to 6 weeks later. This period was considered to be long enough to prevent patients from copying the SQUASH from memory, and short enough to prevent large changes in physical activity levels. Patients who also consented to participate in the validation study (validation group) wore an accelerometer, the ActiGraph™ GT1M monitor (Actigraph™, LLC, Pensacola, Florida, USA), during the two weeks following completion of the second questionnaire. These patients kept a diary in which they noted periods of noncompliance with the Actigraph and/or exceptional activities.

### Physical activity questionnaire

The SQUASH [see additional file 1] was used to assess the physical activity behavior of the study population. It is structured in a way that allows comparing the results to national and international physical activity recommendations. The SQUASH contains questions on commuting activities, leisure-time and sports activities, household activities, and activities at work and school. It consists of three main queries: days per week, average time per day and intensity (effort). In order to keep the questionnaire short and easy to fill in, intensity of household activities and activities at work and school are prestructured into two categories, light or intense, while time spent on activities at work and school is depicted in average time per week.

### Calculation of the activity score per week from the SQUASH

Patients were asked to refer to an average week in the past few months. Using the Ainsworth compendium of physical activities [18,19], activities were assigned a MET value. One MET is defined as the energy expenditure for sitting quietly. Based on the Dutch physical activity guideline [6], activities were subdivided for adults and older adults (up to age 55 and older) respectively into three intensity categories. For adults activities with a MET-value between 2 and < 4 were classified as light, between 4 and < 6.5 as moderate, and ≥ 6.5 as vigorous intensity. For older adults activities between 2 and < 3 MET were classified as light, between 3 and < 5 MET as moderate, and ≥ 5 MET as vigorous intensity. Activities with a MET value lower than 2 were not included because they are considered to contribute negligibly to physical activity level. Based on reported effort in the questionnaire, activities were assigned an intensity score and a total activity score; activity scores for separate questions were calculated by multiplying total minutes of activity by the intensity score.

### Activity monitor

Physical activity was also assessed by means of the ActiGraph™ GT1M activity monitor. This is a compact (3.8 × 3.7 × 1.8 cm), light-weight (27 gr) uniaxial accelerometer, measuring and recording time-varying accelerations ranging in magnitude from approximately 0.05 to twice gravitational acceleration. It is band-limited to a frequency range of 0.25 to 2.5 Hertz, so that normal human motion is detected and motion from other sources rejected. The ActiGraph™ collects and reports physical activity in "counts". Counts are the summation of the accelerations measured during a user-specified time interval (epoch), and represent the intensity of activity in that epoch. In this study, data were collected for each minute during a two-week period. Patients were instructed to wear the monitor during the time they were not asleep, except when showering, bathing or swimming. The monitor was firmly attached to a belt on the waist (sagittal line).

### Calculation of activity from the activity monitor

Activity counts per minute were converted to MET values using the equation published by Freedson et al. [20] (MET value = 1.439008 + (0.000795 * counts/minute)), with cutoff points for the intensity categories consistent with those of the SQUASH. After this conversion, time spent per week in the different intensity categories as well as total time of activity was calculated. Furthermore, mean counts per minute were calculated by dividing the total count over two weeks by the total number of minutes the ActiGraph™ was worn. For purposes of reproducibility of this reference method, the activity level was only calculated for days in which the monitor was worn for 12 hours or longer. Assuming one sleeps for 8 hours a day, this time period represents at least 75% of the available time (16 hr). For purposes of comparability to the reference period of the SQUASH, the monitor had to be worn for at least seven days.

### Statistical analysis

The data were analyzed using the Statistical Package for the Social Sciences (SPSS, Chicago) software (version 14). Descriptive statistics were used to describe the main characteristics of both study populations.

Reliability of the SQUASH was determined by calculating Spearman's correlation coefficient between the activity scores of the separate questions as well as the total activity scores from both administrations. Additionally, a Bland & Altman analysis was performed for the total activity scores [21].

Spearman's correlation coefficients were used to determine relative (or concurrent) validity of the scores on the SQUASH using the Actigraph™ as criterion measure. To this end, the scores of the first SQUASH were used to exclude the possibility of biases resulting from an increased awareness of activity or a learning effect. Spearman's correlation coefficient was assessed between total activity score of the SQUASH and mean counts per minute of the ActiGraph™. Spearman's correlations were also assessed between total time spent in activity, as well as time spent in different intensity categories of physical activity, according to the SQUASH and the ActiGraph™. Additionally, Bland & Altman analyses were performed.

To examine the capability of the SQUASH for categorizing patients according to their physical activity level, the kappa statistic for the tertiles of both activity scores and activity counts as well as the percentage of exact agreement between the tertiles were calculated. This was also performed for the capability of the SQUASH to determine if patients complied with the guidelines of health-enhancing physical activity.

## Results

Demographic characteristics of the study populations are presented in Table 1. Mean age of the patients in the reliability group was 71, with 61.4% female patients. In the validation group the mean age was 70, with 61.5% females. Patients completed the SQUASH for a second time after a mean of 3.7 weeks. No technical errors were encountered during the Actigraph™ registrations.

**Table 1:** Patient characteristics.

	Reliability group *n *= 44	Validity group *n *= 39
Age (years) (mean ± SD)	71 ± 8	70 ± 8
Sex, male/female (n (%))	17 (38.6)/27 (61.4)	15 (38.5)/24 (61.5)
Body Mass Index (kg/m^2^)	26.8 ± 4.5	26.7 ± 3.7
Family status (n (%))		
Alone	12 (27.3)	8 (20.5)
With Partner	32 (72.7)	31 (79.5)
Educational Level (n (%))		
Lower	14 (31.8)	12 (32.4)
Secondary	20 (45.5)	17 (43.6)
Higher	9 (20.4)	7 (18.0)
Other	1 (2.3)	1 (2.6)
Unknown		2 (5.1)
WOMAC* total score (scale 0–100) (mean ± SD)	79.8 ± 18.6	80.8 ± 16.5

Characteristics of the patients included in the reliability and validity group

*WOMAC = Western Ontario and McMaster Universities Osteoarthritis index [27]

Of the reported time (SQUASH), 46% was spent during leisure-time activities, 44% during household activities and 10% at work. Almost no time was spent on commuting activities (Table 2). Assessment of physical activity by means of the SQUASH resulted in substantially more minutes of physical activity in all intensity categories compared to the Actigraph™. Most of the time was spent at low-intensity activities, as assessed by the SQUASH (52%) as well as the Actigraph™ (80%) (Table 3).

**Table 2:** Physical activity of patients in the reliability group and reliability of the SQUASH.

Item	Minutes/week SQUASH-1 n= 44	Activity score SQUASH-1 n = 44	Activity score SQUASH-2 n = 44	Reliability *r*_Spearman_ n = 44
All items together	1694 (1173)	7138 (5577)	5792 (4416)	0.57*
Commuting				
Walking	1 (9)	7 (45)	23 (136)	0.68*
Cycling	0 (0)	0 (0)	36 (171)	-
Activities at work				
Light	142 (488)	284 (977)	202 (564)	0.47*
Intense	27 (126)	136 (632)	68 (452)	0.70*
Household activities				
Light	709 (723)	1443 (1432)	1211 (1378)	0.72*
Intense	31 (82)	164 (425)	213 (540)	0.45*
Leisure time				
Walking	215 (303)	1048 (1515)	588 (665)	0.58*
Cycling	203 (181)	1625 (1529)	1471 (1735)	0.77*
Gardening	113 (145)	889 (1159)	882 (1510)	0.90*
Odd jobs	76 (247)	365 (1209)	345 (1016)	0.57*
Sports	177 (405)	1178 (2825)	753 (2072)	0.84*

Minutes per week spent in different categories of physical activity (mean ± SD) by patients in the reliability group, activity scores from the dual measurements (mean ± SD) and reliability of the total activity scores, as well as reliability of the scores on separate questions of the SQUASH (Spearman correlation coefficient).

* P ≤ .01

**Table 3:** Results of the Bland & Altman method for validity of the SQUASH

	SQUASH-1 (*n *= 39)	Actigraph™ (*n *= 39)	*d*	SE *d*	95% CI
Total	1741 ± 1227	661 ± 475	1060 ± 1052	168	720 – 1400
Low intensity	898 ± 797	530 ± 337	237 ± 784	126	-17 – 491
Moderate intensity	475 ± 651	125 ± 161	238 ± 501	80	76 – 400
Vigorous intensity	368 ± 332	4 ± 9	302 ± 317	51	199 – 405

All times are expressed as minutes of activity per week (mean ± SD).

d = mean difference between time spent in physical activity as assessed by the first administered SQUASH (SQUASH-1) and the Actigraph™.

SE d = standard error of the mean difference.

95% CI = 95% confidence interval of the mean difference between the two measurements.

### Reliability

**Figure 1:**
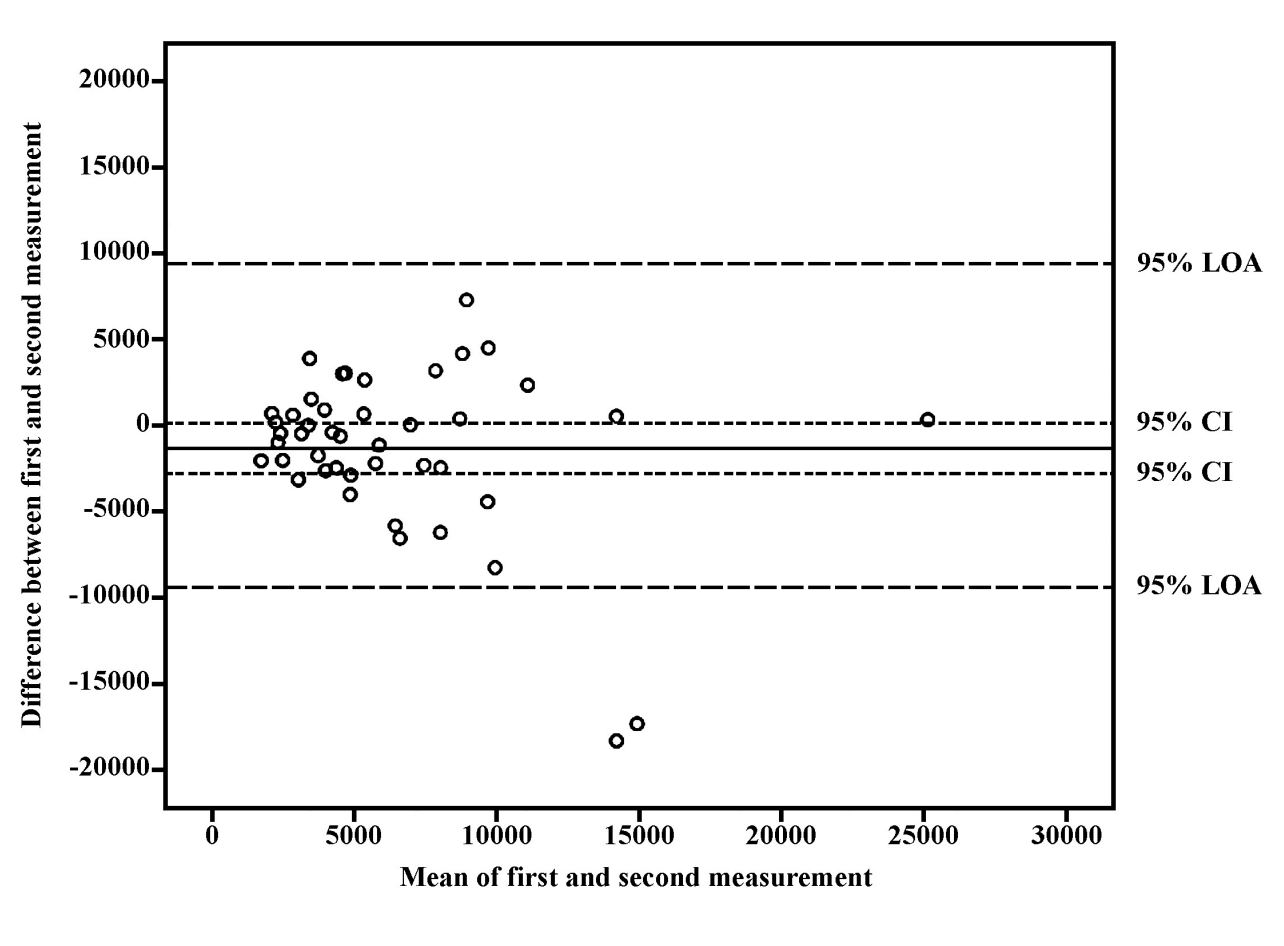
**Bland & Altman graph with limits of agreement(LOA)**. The differences between total activity scores on the first and second SQUASH, plotted against their mean for each patient, together with the 95% confidence interval (CI) and the 95% LOA. Activity score = minutes × intensity.

Spearman's correlation coefficient for the total activity score was 0.57. Reliability for commuting bicycling activities could not be determined because only one of the patients reported this activity on the second questionnaire. For the other, separate questions the Spearman correlation coefficients ranged from 0.45 to 0.90, with a mean value of 0.61. Intense household activity was the least reliable, while gardening was the most reliable physical activity (Table 2). Reliability within the low-, moderate- and vigorous-intensity categories was 0.54, 0.55 and 0.85 respectively. Bland and Altman analysis for the total activity score showed no significant difference between the two measurements, with most observations staying at the 0 ± 1.96 SD range and within the 95% limits of agreement (Fig. 1), indicating no systematic bias between measurements.

### Relative validity

**Figure 2:**
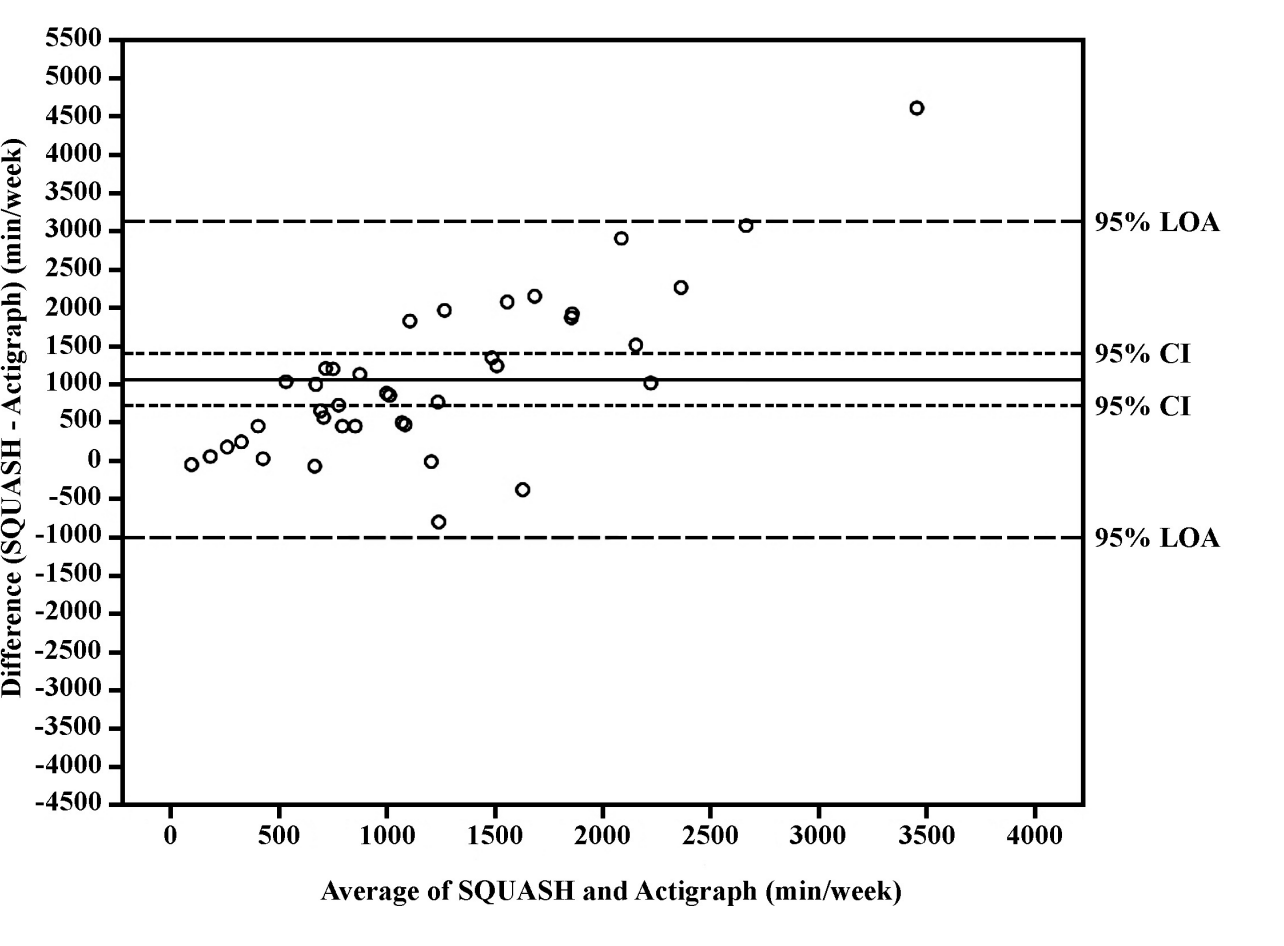
**Bland & Altman graph with limits of agreement(LOA)**. The differences between total minutes of physical activity per week as assessed by means of the SQUASH and the Actigraph™, plotted against their mean for each patient, together with the 95% confidence interval (CI) and the 95% LOA.

Spearman's correlation coefficient between total activity score and mean counts per minute was 0.67 (P = 0.01). The Spearman's correlation coefficient between total minutes of activity as assessed by the SQUASH and the Actigraph™ was 0.56 (P = 0.01), while this coefficient was 0.20 (P = 0.22) for time spent in light intensity activities, 0.40 (P = 0.40) for time spent in moderate intensity activities and 0.35 (P = 0.03) for time spent in vigorous intensity activities. Bland & Altman analysis showed that the total volume of physical activity (Fig. 2), as well as time spent in moderate intensity and vigorous intensity physical activity (Table 3) was systematically higher when assessed by means of the SQUASH, compared to the ActiGraph™. Furthermore data showed heteroscedacity, which remained after log transformation.

When the tertiles of the activity scores were compared with the tertiles of the activity counts the exact agreement was 67% and the weighted kappa 0.50. With respect to compliance with the guidelines the exact agreement was 49% and the weighted kappa 0.12.

## Discussion

Despite recognized benefits of regular physical activity, little is known about the physical activity behavior of patients after THA. We therefore examined the measurement properties of the SQUASH as a tool to provide more insight into this behavior, as this short, self-reported physical activity questionnaire has been shown to be a fairly reliable and valid tool to assess the physical activity behavior of the general Dutch adult population [10].

The Spearman correlation for overall reliability of the SQUASH in our study was 0.57. As, to our knowledge, this is the first study to assess the measurement properties of a physical activity questionnaire in patients after THA, we are unable to compare our results to studies in a similar population. However, this overall reliability of the SQUASH is almost identical to the reliability of 0.58 found in the study by Wendel-Vos [10], assessing the measurement properties of the SQUASH in a population of 50 healthy adults (mean age 44 ± 6 yr). Although our study design was largely identical to that of Wendel-Vos, if we are to compare our results to those of the original study into the reliability and validity of the SQUASH it must be stated that the Wendel-Vos study differed from ours in that participants first completed the SQUASH for a second time before the Actigraph™ readings were performed. This was done to prevent a possible influence on reliability due to an increased awareness about physical activity, which might occur when the Actigraph™ is worn between the two measurements of the SQUASH. Reliability is also consistent with the reliability of other physical activity questionnaires, validated by means of an accelerometer in adult populations. In a review of seven physical activity questionnaires, validated with accelerometers in adults, reliability varied between 0.34 and 0.89 [22]. Also, a study into the reliability and validity of the International Physical Activity Questionnaire (IPAQ), which is comparable to the SQUASH but was developed for cross-national monitoring of physical activity, showed a Spearman correlation coefficient for the short forms of the IPAQ ranging from 0.25 to 0.88, with a pooled reliability of 0.76 [23]. The reliability found in our study is thus comparable with reliabilities found in other physical activity questionnaires. Furthermore, Bland and Altman analysis showed no systematic bias on total activity scores between test and retest.

The total activity score on the SQUASH correlated significantly with the mean activity counts per minutes measured by the Actigraph™ (*r*_Spearman _= 0.67). The total minutes of activity as assessed by the SQUASH and the Actigraph™ also correlated significantly (*r*_Spearman _= 0.56). Hence the SQUASH can explain 31% of the total variation in physical activity. When comparing the tertiles of activity scores with the tertiles of activity counts, exact agreement was 67%, which is fair to good. The weighted kappa was 0.50, representing fair agreement. The relative validity of the SQUASH in our study is higher than that found in the study by Wendel-Vos, showing a Spearman correlation coefficient between total activity score and accelerometer readings of 0.45. Comparison of the tertiles of the activity score with tertiles of the activity counts in their study showed an exact agreement of 46% and a weighted kappa of 0.30, which are lower values than those found in our study. In the Sallis review of seven physical activity questionnaires [22], validity correlations ranged from 0.14 to 0.53. The IPAQ short forms showed validity ranging from -0.12 to 0.57, with a pooled Spearman correlation coefficient of 0.33 [23]. It can therefore be concluded that the validity found in our study lies in the upper range of validity found in other questionnaires validated with an accelerometer in adult populations. However, consideration should be given to the sizeable systematic bias between the scores on the SQUASH and the Actigraph™ readings. This systematic bias may be the result of overestimating physical activity level by the SQUASH, as people tend to overestimate their physical activity level [22]. At the same time the Actigraph™ may have underestimated physical activity level. The Actigraph™ is a uniaxial accelerometer for vertical movement and is relatively insensitive to physical activities that require little vertical movement. When positioned on the waist activities such as cycling or activities involving large upper-body movement may be underestimated. Additionally, the accelerometer is not waterproof and therefore cannot be worn during activities such as swimming. Since in our study 21% of patients reported swimming and 77% cycling as part of leisure-time activities, this will have led to an underestimation of physical activity by the Actigraph™. The systematic bias may also reflect true variations in participants' physical activity levels. Since the SQUASH asks patients to recall physical activity during an average week in the past months, this timeframe was not identical to the period of time used to acquire the accelerometer data. Furthermore, to estimate the energy expenditure spent in physical activity the activity counts as obtained by the Actigraph™ have to be transformed into MET values. To do this, regression equations have been developed from studies under laboratory as well as field conditions [20,24,25]. In line with the study of Wendel-Vos we used the Freedson equation to transform activity counts into MET values. As this regression equation was developed under laboratory conditions, it may not be valid under the "field conditions" of our study as it particularly has been shown to underestimate moderate-intensity activity [26]. Additionally, the regression equation was developed in adults and may not be appropriate for older adults. However, to our knowledge, there are no regression equations specifically for older adults. This may be another factor accounting for the differences found in our study in terms of time spent in the different intensity categories between the physical activity questionnaire and the accelerometer.

## Conclusion

The SQUASH can be considered to be a fairly reliable tool to assess the physical activity behavior of patients after primary THA, while the validity is comparable to those of other physical activity questionnaires. As it is short and easy to fill in, it can be used to assess the physical activity of patients after primary THA with minimal cost and burden to the subjects. However, using the Actigraph™ as a criterion measure considerable systematic bias was found between the scores on the SQUASH and the Actigraph™ readings. Therefore more research is needed to assess the validity of the SQUASH using other objective criteria and cut-points appropriate for the population under study.

## Competing interests

The authors declare that they have no competing interests.

## Authors' contributions

RW and MS conceived the idea of the manuscript, conducted the study and contributed in drafting the manuscript. JWJK and IvdA-S contributed in data collection, as well as drafting and revising the manuscript. JWG, WZ, SKB, IvdA-S, SKB and GCW W-V contributed in drafting and revising the manuscript. JJAM van R contributed with institutional liaisons as well as drafting and revising the manuscript. All authors have read and approved the final manuscript.

## Pre-publication history

The pre-publication history for this paper can be accessed here:



## Supplementary Material

Additional file 1 **Short questionnaire to assess health-enhancing physical activity (SQUASH).** This document presents the short questionnaire to assess health-enhancing physical activity (SQUASH). Click here for file
