# Supplementary material for: Reliability and validity of the short questionnaire to assess health-enhancing physical activity (SQUASH) in patients after total hip arthroplasty
Source: BMC Musculoskelet Disord. 2008 Oct 17;9:141. doi: 10.1186/1471-2474-9-141 (PMC2576250; doi:10.1186/1471-2474-9-141)
Supplement: Supplementary file 1 [file 1471-2474-9-141-S1.doc]

## Appendix A: *The short questionnaire to assess health enhancing physical activity (SQUASH)*

Think about an average week in the past months. Please indicate **how many days per week** you performed the following activities, how much time **on average** you were engaged in this, and (if applicable) how strenuous this activity was for you?

| **COMMUTING ACTIVITIES (round trip)** | **days per *week*** | **average time per *day*** | **Effort (circle please)** |
|---|---|---|---|
| **Walking to/from work or school** | days | hour minutes | slow/moderate/fast |
| **Bicycling to/from work or school** | days | hour minutes | slow/moderate/fast |
| Not applicable | | | |

| **LEISURE TIME ACTIVITIES** | **days per *week*** | **average time per *day*** | **Effort (circle please)** |
|---|---|---|---|
| **Walking** | days | hour minutes | slow/moderate /fast |
| **Bicycling** | days | hour minutes | slow/moderate /fast |
| **Gardening** | days | hour minutes | light/moderate /intense |
| **Odd jobs** | days | hour minutes | light/moderate /intense |
| **Sports** (please write down yourself) *e.g., tennis, fitness, skating, swimming, dancing* | | | |
| 1. .............................. | days | hour minutes | light/moderate /intense |
| 2. .............................. | days | hour minutes | light/moderate /intense |
| 3. .............................. | days | hour minutes | light/moderate /intense |
| 4. .............................. | days | hour minutes | light/moderate /intense |

| **HOUSEHOLD ACTIVITIES** | **days per *week*** | **average time per day** |
|---|---|---|
| **Light household work** | days | hour minutes |
| (cooking, washing dishes, ironing, child care) | days | hour minutes |
| **Intense household work** (scrubbing floor, walking with heavy shopping bags) | | |

| **ACTIVITY AT WORK AND SCHOOL** | **average time per *week*** |
|---|---|
| **Light work** | hour minutes |
| (sitting/standing with some walking, e.g., a desk job) | hour minutes |
| **Intense work** (regularly lifting heavy objects at work) | |
| Not applicable | |
